# Dose, exposure, and treatment regimen of intravenous immunoglobulin G in multifocal motor neuropathy

**DOI:** 10.3389/fneur.2024.1478419

**Published:** 2024-11-06

**Authors:** Zhaoyang Li, Stefan Roepcke, Ryan Franke, Leman Yel

**Affiliations:** ^1^Global Clinical and Translational Sciences, Plasma-Derived Therapies, Takeda Development Center Americas, Inc., Cambridge, MA, United States; ^2^Pharmacometrics, Simulations Plus, Inc., Buffalo, NY, United States; ^3^Clinical Pharmacology, Cognigen Division of Simulations Plus, Inc., Buffalo, NY, United States; ^4^Global Clinical Sciences, Takeda Development Center Americas, Inc., Cambridge, MA, United States

**Keywords:** intravenous immunoglobulin, multifocal motor neuropathy, immunoglobulin, population pharmacokinetic modeling, dose-exposure relationship

## Abstract

**Introduction:**

Intravenous immunoglobulin (IVIG) is the only approved treatment for multifocal motor neuropathy (MMN), a rare, chronic, immune-mediated demyelinating neuropathy. There is a significant gap in understanding of the role of serum immunoglobulin G (IgG) levels in the efficacy of IVIG in affected patients. We aimed to characterize the interplay between dose and exposure of IVIG and the effects of patient factors on individual variabilities.

**Methods:**

Serum IgG trough concentration data from a phase 3, randomized, double-blind, placebo-controlled, crossover trial of IVIG 10% in 44 patients with MMN (NCT00666263) were analyzed using fit-for-purpose population PK modeling. Patient factors were tested as covariates, and IgG PK profiles following various dosing regimens were simulated.

**Results:**

Serum IgG levels, with significant inter-patient variability, correlated with dose and treatment interruptions at the individual patient level. Simulated data for various dosing regimens (0.4–2 g/kg once every 1–4 weeks [Q1–4W]) revealed that more frequent dosing provided more stable IgG levels than less frequent dosing, and dose splitting over multiple days had no significant effects on PK.

**Discussion:**

In patients with MMN, stable dosing and consistent serum IgG levels are crucial to avoid negative responses owing to treatment interruptions. Dosing intervals more frequent than Q4W may alleviate periodic symptom deterioration. Dose splitting potentially offers flexibility for patients requiring large volumes of IVIG without negatively affecting serum IgG PK, while maintaining treatment efficacy. Variability in serum IgG levels between patients suggests that individualizing IVIG treatment regimens and target IgG levels may play a key role in managing MMN.

## Introduction

Multifocal motor neuropathy (MMN) is a chronic, immune-mediated, demyelinating neuropathy that causes slowly progressive, asymmetric, distal limb weakness (most commonly of the arms) (1). MMN is a rare condition, with a study in the Netherlands estimating disease prevalence as 0.6 per 100,000 individuals (2, 3). Affected patients experience a gradual progression of symptoms, which, without remission, may lead to functional disability that compromises simple daily activities such as writing, washing, or dressing (4–6).

While there is currently no cure for MMN, symptoms can be stabilized with early diagnosis and treatment (5, 7, 8). A characteristic hallmark of MMN is conduction block, defined as the inability of motor nerves to propagate action potentials in affected areas (9, 10). This is thought to be due to the production of antibodies against glycolipid GM1, causing functional disturbances and disruption of nerve conduction (9, 10). Immunoglobulin (Ig) G, administered intravenously (IVIG), is thought to interfere with antibody production by binding to B-cell receptors or inducing inhibitory receptors (9). IVIG is currently the only approved treatment in the USA and Europe for induction and maintenance therapy in adults with MMN, and has shown beneficial long-term effects on muscle strength and limb disability (11, 12). The recommended dose and treatment regimen for IVIG varies from patient to patient, based on body weight and their clinical status (11, 12). Current clinical research aims to identify optimal Ig maintenance dosing regimens, including for different routes of administration (e.g., facilitated subcutaneous Ig treatments containing hyaluronidase) and to assess the long-term effects of Ig treatment, but studies are limited in size owing to the rarity of the condition (typically *n* < 20) (8, 13–15).

Serum IgG concentration has been demonstrated to be a valuable marker for treatment effectiveness of Ig therapies in primary immunodeficiency diseases (PIDs), where the relationship between dose, pharmacokinetics (PKs), and clinical outcomes is well understood (16). However, in neuroimmunological diseases, such as chronic inflammatory polyradiculoneuropathy (CIDP) and MMN, the understanding of this relationship is still lacking.

Pharmacokinetic and pharmacokinetic-pharmacodynamic (PK-PD) modeling and simulations have evolved into an integral part of drug development, allowing regulatory and therapeutic questions to be addressed and informing development strategy and decision-making (17, 18). In particular, PK and PK-PD modeling has been used as an effective tool to determine and optimize dosing regimens for phase 1 to phase 3 clinical trials, informing labeling and guiding therapy use in clinical settings (19). For IgG specifically, there have been multiple studies that have successfully employed PK modeling to simulate IgG levels following interventions in patients with PIDs, enabling the optimization of dosing regimens and the identification of intrinsic and extrinsic factors that may significantly affect IgG levels following treatment (20–24). However, to date, modeling studies have generally utilized more robust PK sampling (both dense and sparse at various time points) to develop models, and in most clinical settings, it is not feasible to collect these types of samples in patients with conditions such as MMN. No modeling studies have yet been published describing the interplay between IVIG dose and serum IgG concentration through PK modeling, with only sparse serum trough samples collected from patients with MMN. Modeling may not only provide a tool to predict individual patient IgG exposure profiles during therapy and help to optimize treatment in different patient populations, but more widely, to support dose selection and the design of future trials in this disease (25). We undertook both PK and PK-PD modeling to gain insights into the PK profiles of IgG in MMN (described herein) and the relationships between IVIG dose, serum IgG concentrations, and clinical efficacy (the latter described in a separate publication (26)). Both aspects are equally important in addressing the knowledge gaps regarding the treatment of MMN with Ig therapies.

The main aims of this analysis were to characterize the pharmacokinetics of serum total IgG following administration of IVIG containing 10% IgG (IVIG 10%) to patients with MMN, including the typical time course in serum total IgG concentration, variability between and within patients, and clinically important predictors of differences in exposure. To achieve this, a population PK (popPK) model was developed to describe serum total IgG PK following IVIG 10% administration (describing both endogenous production and exogenous administration of IgG), and potential covariate effects on individual PK parameters variability were evaluated as a key part of the model development. Following modeling, simulations were performed for various IVIG dosing regimens of clinical relevance for patients with MMN and the associated serum total IgG PK profiles.

## Materials and methods

### Data sources and handling

**Figure 1:**
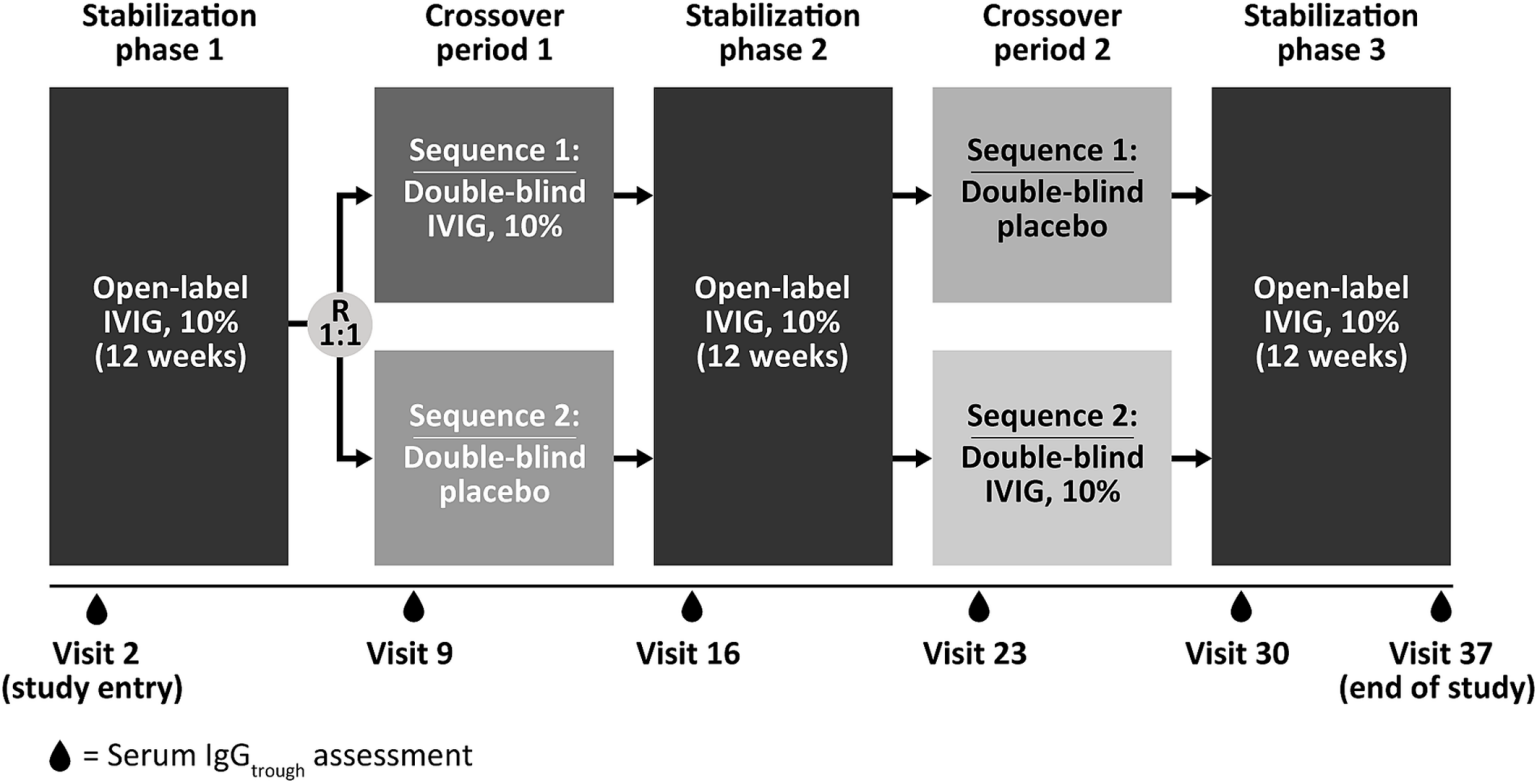
Study design of the phase 3, randomized, double-blind, placebo-controlled, crossover trial of IVIG 10% in 44 patients with multifocal motor neuropathy (NCT00666263) used as the data source for serum trough concentration in the current analysis. Patients included in the study had a diagnosis of definite or probable MMN and were on a stable IVIG 10% regimen of 0.4–2 g/kg body weight every 2–5 weeks (and had been for at least 3 months) at enrolment. During the 60-week study, dosing regimens for IVIG 10% were in the range 0.4–2 g/kg/infusion cycle, divided over 1–5 consecutive days, with a once every 2-, 3-, or 4-weekly dosing cycle. IgG_trough_, immunoglobulin G trough concentration; IVIG 10%, intravenous immunoglobulin containing 10% IgG; R, randomization.

Sparse serum trough concentration data were extracted from a phase 3, randomized, double-blind, placebo-controlled, crossover trial of IVIG 10% (GAMMAGARD LIQUID, Baxalta US Inc., a member of the Takeda group of companies, Cambridge, MA, USA; Kiovig, Takeda Manufacturing Austria AG, Vienna, Austria) in 44 adult patients with MMN (NCT00666263) (11, 12, 27). Full details of the methodology and clinical results have been previously published (27). In brief, patients included in the phase 3 trial had a diagnosis of definite or probable MMN and were on a stable IVIG 10% regimen of 0.4–2 g/kg body weight every 2–5 weeks (and had been for at least 3 months) at enrollment (27). After an initial 12-week clinical stabilization period on open-label IVIG 10%, patients were then randomized (1:1) to one of two treatment sequences of 12 weeks of IVIG 10% or placebo (first double-blinded crossover period), followed by a second 12-week stabilization period of open-label IVIG 10% to avoid carryover effects (27). This was then followed by a second 12-week double-blinded crossover period of IVIG 10% or placebo, and a final 12-week stabilization period (open-label IVIG 10%) (27). If patients experienced significant deterioration during crossover periods, they were permitted to move to the next IVIG 10% stabilization period early (referred to as accelerated switch). The design of the phase 3 trial is summarized in Figure 1. During the 60-week study, dosing regimens for IVIG 10% were in the range 0.4–2 g/kg/infusion cycle, divided over 1–5 consecutive days, with a once every 2-, 3-, or 4-weekly dosing cycle (27). Serum samples for PK analysis of total IgG concentration were collected at screening, prior to dose administration at the beginning of each stabilization or crossover period, at the end of the final stabilization period, and at the end of the study.

Individual patient data extracted for the popPK analysis data set included serum total IgG concentrations, dosing, treatment (IVIG 10% or placebo) and PK sampling information, demographics, clinical laboratory values, and details of other clinical covariates.

As this modeling study used preexisting, anonymized data from a previous study in which informed consent was obtained (27), no ethics approval or consent for publication was required.

### Data analysis

#### Exploratory analysis of dose–serum IgG exposure correlation

A preliminary exploratory data analysis was performed to assess trends in the data. The correlation between IVIG 10% dose and changes in serum total IgG trough levels during the switch from stable IVIG treatment to placebo, or vice versa, was evaluated. Serum IgG trough levels were assessed at the beginning of each stabilization phase and crossover period, and at the end of the study. The changes in absolute values and percentage changes were calculated relative to baseline values at study entry and the prior visit.

#### Population PK modeling of serum IgG levels

Serum IgG trough concentration–time data were analyzed using a nonlinear mixed-effects modeling approach using NONMEM version 7.3.0 (ICON, Hanover, NH, USA) (28). All data analyses and presentations were performed using SAS version 9.4 (SAS Institute Inc. NC, USA) (29), R version 3.4.3 (R Foundation, Vienna, Austria) (30), and KIWI version 4 (Cognigen, a division of Simulations Plus, NY, USA) (31).

Patient demographic factors, including age, sex, body weight, body mass index (BMI), lean body mass (LBM), and creatinine clearance, were evaluated as potential covariates for inclusion. Baseline covariate values were used for the purposes of this modeling analysis because it was assumed that covariate values would remain essentially constant throughout the study period.

#### Simulations

Using the final model, simulations were performed to predict the serum total IgG concentration profiles under the following dosing regimens: 0.4, 0.8, and 1 g/kg every 2 weeks (Q2W); 1 and 2 g/kg every 3 weeks (Q3W); 0.4, 0.8, 1, and 2 g/kg every 4 weeks (Q4W); and 2 g/kg Q4W split into four daily infusions.

## Results

### Analysis data set and patient population

Following exclusion of any duplicate records and outliers, the full PK analysis data set included a total of 309 serum IgG concentration records from 44 patients. Baseline clinical covariates for these 44 patients are summarized in Supplementary Table S1. In brief, patients had a mean (standard deviation [SD]) age of 51.7 (10.3) years and a BMI of 27.9 (4.1) kg/m^2^, and 72.7% of patients were male. The mean (SD) total IgG dose at baseline was 1 (0.5) g/kg, with 18.2% of patients receiving doses Q2W, 20.5% of patients receiving doses Q3W, and 61.4% of patients receiving doses Q4W.

### Exploratory analysis of dose-serum IgG exposure correlation

**Figure 2:**
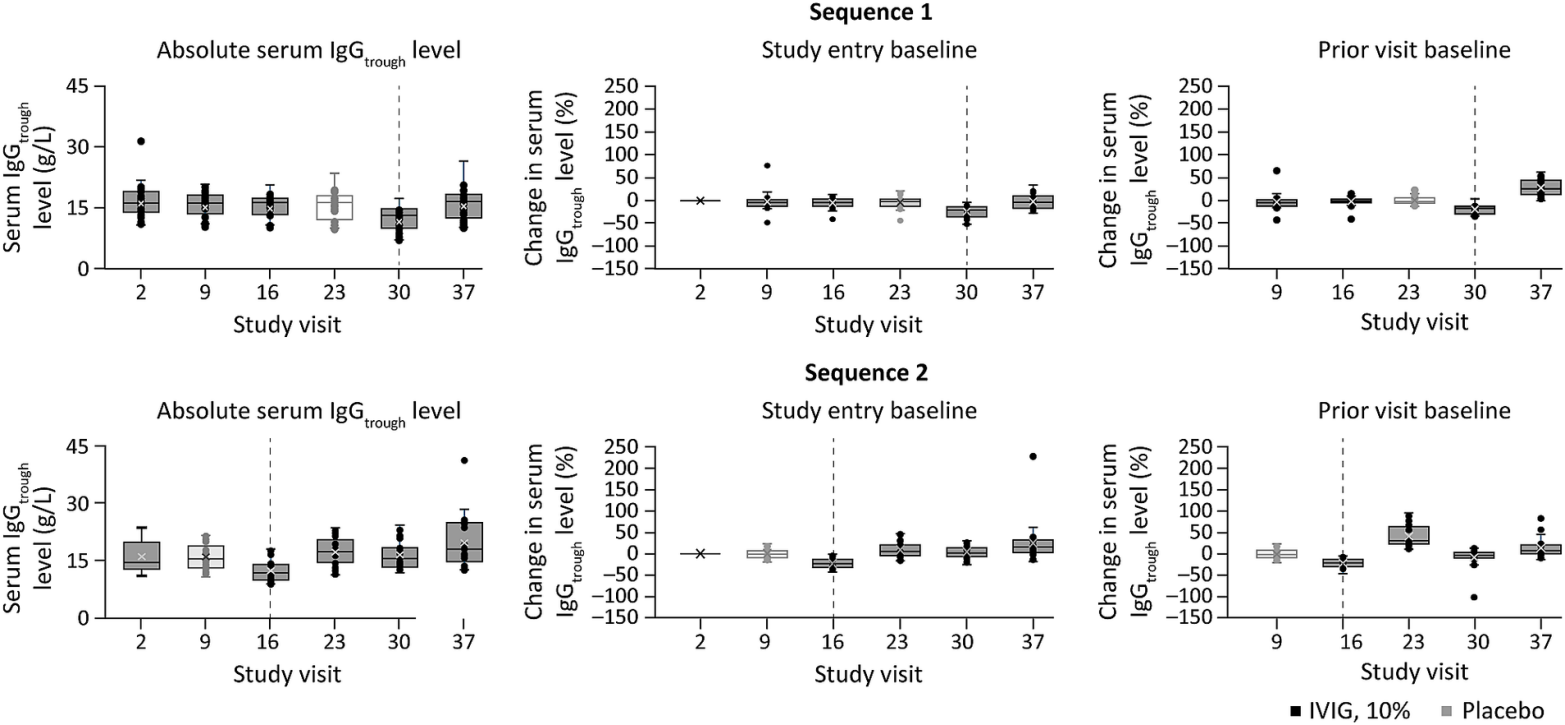
Percent change in serum trough IgG level at study visits, relative to baseline or the prior visit level^a^, and stratified by treatment sequence (exploratory dose–serum IgG exposure analysis). ^a^Percent change in serum trough IgG level was calculated relative to either the value at study entry or at the prior visit (middle and right panels, respectively). Dotted lines indicate point of treatment switch. IgG, immunoglobulin G; IgG_trough_, trough immunoglobulin G; IVIG 10%, intravenous immunoglobulin containing 10% IgG.

**Figure 3:**
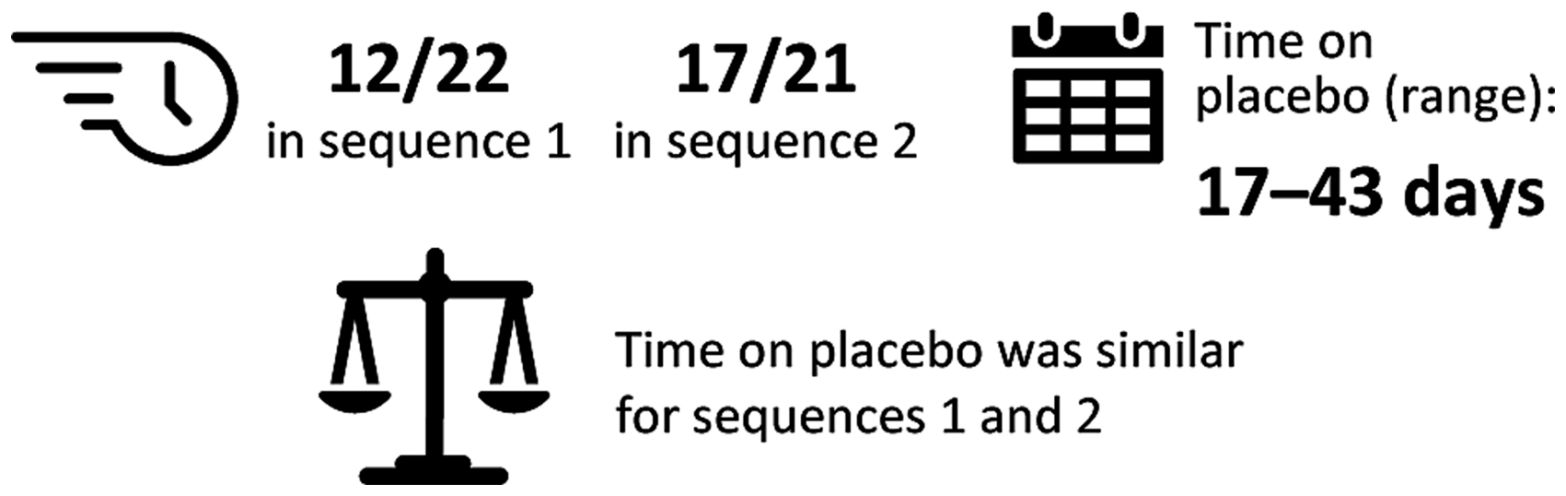
Summary of accelerated switchers^a^ (exploratory analysis). ^a^Accelerated switchers were patients who were permitted to move to the next IVIG 10% stabilization period early if they experienced deterioration of their condition. One patient (2.4%) switched from blinded to open-label IVIG 10%, but did not switch during placebo treatment. IgG, immunoglobulin G; IVIG 10%, intravenous immunoglobulin containing 10% IgG.

In the exploratory dose–exposure correlation analysis, serum trough IgG levels were highly variable among patients with MMN and were observed to significantly decrease following a switch from IVIG 10% to placebo in both treatment sequences (*p* < 0.01; Figure 2). The majority of patients (67.4%) had an accelerated switch back to IVIG 10% (i.e., a shorter placebo period than defined in the study protocol; Figure 3). Among those accelerated switchers, monthly IVIG 10% dose received during the scheduled blinded placebo period increased compared with the previous stable IVIG 10% dose (mean increases of 42.2 and 47.4% for sequence 1 [IVIG then placebo] and sequence 2 [placebo then IVIG], respectively). As expected, in these patients, median trough IgG levels were slightly higher than in those who completed placebo treatment. Across the whole study, median (range) serum trough IgG levels were 16.4 (9.9–41.0) g/L for stable IVIG periods and 12.4 (7.0–18.1) g/L for placebo periods. A significant correlation was observed between the total administered dose of IVIG 10% and serum trough IgG levels (*p* < 0.01) for both treatment sequences.

### Population PK modeling of serum IgG levels

Serum total IgG concentration-time profiles following IVIG 10% administration were adequately described using the final popPK model (Table 1; Supplementary results).

**Table 1:** Parameter estimates for the final popPK model of IVIG 10%.

Parameter	Final parameter estimate		Magnitude of variability	
	Population mean	%RSE	Final estimate (%CV)	%RSE
V1, mL	6,590	8.42	18.0	48.7
Exponent of LBM (LBM/56.54) for V1	2.23	15.7		
CBASE, mg/mL	11.1	3.21	18.2	20.1
K_EL_, L/h	0.00241	7.51	NE	NA
Residual variability	0.00905	17.1	9.51	NA
Minimum OFV = 711.896				

%CV, percentage coefficient of variation; %RSE, relative standard error; CBASE, latent model-derived parameter for IgG concentration in the absence of treatment; IgG, immunoglobulin G; IVIG 10%, intravenous immunoglobulin containing 10% IgG; K_EL_, elimination rate constant; LBM, lean body mass; NA, not applicable; NE, not estimated; OFV, objective function value; popPK, population pharmacokinetic; V1, volume of distribution.

### Model-based simulations

**Figure 4:**
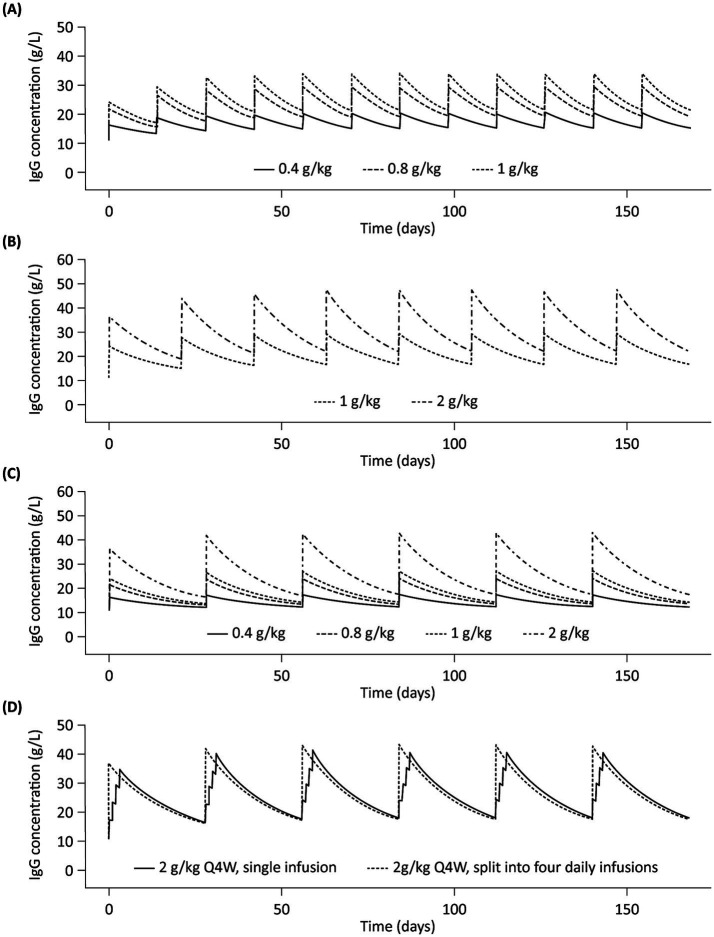
Simulated profiles of serum IgG concentrations in a typical patient with multifocal motor neuropathy during 6 months of IVIG 10% treatment with (A) Q2W dosing, (B) Q3W dosing, (C) Q4W dosing, and (D) 2 g/kg Q4W split into four daily infusions. IgG, immunoglobulin G; IVIG 10%, intravenous immunoglobulin containing 10% IgG; Q2W, every 2 weeks; Q3W, every 3 weeks; Q4W, every 4 weeks.

**Figure 5:**
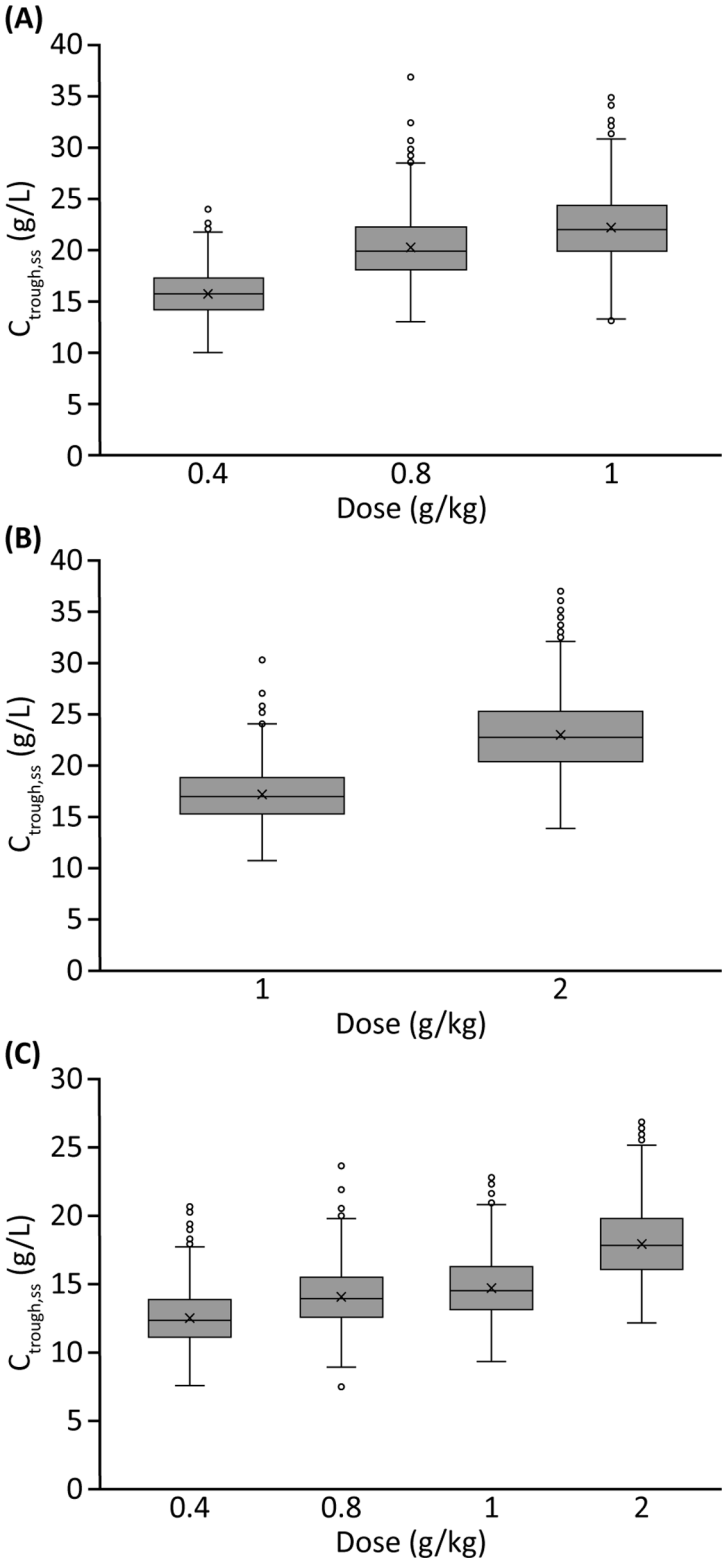
Simulated trough serum IgG concentrations at steady-state for different IVIG 10% dosing scenarios^a^: (A) Q2W dosing, (B) Q3W dosing, and (C) Q4W dosing. ^a^Monthly doses varied across dosing scenarios, e.g., 1 g/kg corresponds to a monthly dose of 2 g/kg with Q2W dosing, 1.33 g/kg with Q3W dosing, and 1 g/kg with Q4W dosing. C_trough,ss_, trough IgG concentration at steady-state; IgG, immunoglobulin G; IVIG, intravenous immunoglobulin G; Q2W, every 2 weeks; Q3W, every 3 weeks; Q4W, every 4 weeks.

Summary profiles of simulated IgG concentrations for Q2W, Q3W, and Q4W dosing scenarios, assuming typical values of model parameters and median values of body weight and LBM, are presented in Figure 4. Simulated trough IgG concentrations at steady-state (during dosing intervals) for each scenario are presented in Table 2 and Figure 5. Overall, steady-state trough serum IgG levels appeared to increase proportionally with dose across all regimens (Q2W, Q3W, and Q4W). There was some degree of accumulation with Q2W and Q3W dosing, with steady-state reached after approximately four doses and three doses, respectively. With the Q4W dosing regimen, there was almost no observable accumulation, and trough levels almost reached estimated baseline pretreatment levels for all but the 2 g/kg dose. At higher doses, splitting of the dose into four daily infusions did not markedly impact the PK concentration–time profiles for IVIG 10%.

**Table 2:** Simulated trough serum IgG concentrations during IVIG 10% dosing intervals at steady-state for different dosing scenarios.

	Dose^b^			
	0.4 g/kg	0.8 g/kg	1 g/kg	2 g/kg
C_trough,ss_ (g/L)^a^, dosing Q2W	15.6 (14.8)	20.0 (15.3)	21.9 (14.9)	–
C_trough,ss_ (g/L)^a^, dosing Q3W	–	–	17 (15.2)	22.8 (15.8)
C_trough,ss_ (g/L)^a^, dosing Q4W	12.4 (16.7)	13.9 (15.6)	14.6 (15.4)	17.8 (14.6)

^a^Geometric mean (%CV) values. ^b^Monthly doses will vary across dosing scenarios, e.g., 1 g/kg corresponds to a monthly dose of 2 g/kg with Q2W dosing, 1.33 g/kg with Q3W dosing, and 1 g/kg with Q4W dosing. %CV, coefficient of variation expressed as a percentage; C_trough,ss_, trough immunoglobulin G concentration at steady-state; IgG, immunoglobulin G; IVIG 10%, intravenous immunoglobulin containing 10% IgG; Q2W, every 2 weeks; Q3W, every 3 weeks; Q4W, every 4 weeks.

## Discussion

MMN is a rare condition, with an estimated prevalence of 0.6 per 100,000 individuals (2, 3); and unlike in some other disease areas, the understanding of the mechanism of action of IVIG in MMN and the role of IgG levels in IVIG treatment efficacy are yet to be elucidated. In addition, information on the associated dose-exposure (PK) and dose-exposure-response (PK-PD) relationships in this population remains hugely limited. In this study, we aimed to characterize the dose-exposure relationship of IVIG.

To characterize the systemic exposure and PK of IgG in serum and understand the relationship between IVIG 10% dose and IgG serum concentrations, an exploratory dose–serum IgG exposure correlation analysis was conducted, followed by a popPK modeling analysis based on serum trough levels of total IgG in patients with MMN. The exploratory analysis of the phase 3 study data showed that IVIG 10% dosing and serum IgG levels were significantly correlated. Patients often responded negatively to treatment interruption and required a higher IVIG 10% dose after resuming therapy, highlighting the importance of maintaining stable IVIG 10% treatment in patients with MMN. In addition, serum IgG trough levels were highly variable between patients, indicating that individualizing IVIG treatment regimens and target IgG levels may play a key role in the management of this condition.

The analysis in this study is the first of its type to describe the systemic exposure and PK of IgG following IVIG 10% administration in patients with MMN, and was established based on sparse serum trough concentrations of total IgG from a randomized phase 3 study (27). The analysis also accounted for endogenous IgG production in the model, an advantage over previously reported analyses for IgG in wider disease areas, such as PIDs (20, 32). In the current study, LBM was shown to have a significant impact on the volume of distribution parameter V1, which was expected, because it is a function of body weight that corrects for body space poorly accessible to IgG, and may be used to describe increases in clearance with body size while accounting for body composition and scaling principles (33). However, while LBM was also correlated with sex and body weight, there was no significant relationship between body size and any exposure metric. This may be due to multiple factors, including protocol-permitted flexibility in dose and administration frequency, body weight-based dosing, and the sparse PK data available from the phase 3 study. Inclusion of additional data in the model would likely improve the precision of estimation of body-size effects and should be considered for future trial designs.

Dosing frequency represents a critical consideration in maintaining IgG concentrations in MMN because patients likely have their own protective thresholds for serum IgG, as in other conditions such as PIDs. The IgG level necessary for disease control and symptom management requires careful and individually tailored consideration during the treatment optimization process for MMN (34). Our model-based simulations for the various clinical dosing scenarios showed some degree of accumulation in serum IgG levels, and that steady-state IgG concentrations were achieved within 3–4 doses for the Q2W and Q3W dosing regimens, which was expected based on the half-life of IgG. In contrast, there was almost no accumulation with a Q4W dosing regimen, and pre-dose trough levels almost reached, or returned to, baseline concentrations for all but 2 g/kg dose levels. This indicates that, while Q4W dosing might be more convenient, the lower pre-dose trough IgG levels and larger fluctuation of IgG levels, as compared with shorter treatment intervals, may manifest in symptom worsening toward the end of dosing intervals when IgG levels are at their lowest. In clinical practice, the doses and treatment frequencies are often determined empirically owing to a paucity of dose–exposure–response data and ‘wearing-off,’ i.e., the cyclic or periodic occurrence of clinical deterioration before the next dose is due (35–38).

Relatively high doses of IVIG (e.g., 2 g/kg/month) are common in patients with MMN, necessitating large dose volumes and long infusion durations (11, 12). At high doses (e.g., 2 g/kg), when a large dose volume is required, splitting the dose over multiple days can be considered in clinical settings. Our analysis has shown that, in one dosing course, splitting doses over multiple days did not markedly affect PK concentration–time profiles and would not be expected to have a significant or clinically meaningful impact on patient symptoms, potentially offering flexibility for patient dosing.

While there is a paucity of dose–exposure–response studies in the literature regarding IgG in patients with MMN, a small study (*n* = 23) has previously evaluated the relationship between total IgG levels and response to treatment after 5 days in IVIG-naive individuals with MMN receiving their first dose of 2 g/kg of IVIG (39). The pretreatment IgG levels observed (13.6 g/L; %CV: 35%) were consistent with the CBASE values reported here (11.1 g/L; %CV: 18.2%) (39). The authors also confirmed that IVIG PK varies in patients with MMN, which may be associated with clinical response, although the underlying cause of differences in IgG remained unknown (39). Our current analysis provided a more in-depth look into the serum PK of IgG based on a larger and more robust data set, and explored a broader range of patient factors for their potential effects on inter-patient variabilities. This dose-exposure analysis, along with an associated exposure-response (PK-PD) analysis (26), will set the foundation for further understanding of the mechanisms of action of IVIG in patients with MMN.

It is noteworthy that this analysis may be limited by the relatively sparse PK data in the phase 3 study in comparison with the generally larger data sets in trials with more common indications. Given that only serum trough PK data were available, the characterization of full concentration–time profiles for IgG following intravenous IVIG 10% administration may be limited, particularly during the distribution phase. More frequent and robust PK sampling and the associated patient burden represent substantial challenges when conducting clinical studies in individuals with rare conditions, especially with the majority of patients with MMN being young and having more active lifestyles. While the relative sparsity of PK data may present limitations, the crossover nature of the study provided comparative patient data versus placebo, increasing the robustness of the analysis. Additionally, given that all patients were on stable IVIG treatment at study entry, serum IgG concentrations in the absence of treatment were not available, potentially affecting the accuracy of the estimated endogenous production of IgG in the model. Overall, the inclusion of more comprehensive IVIG PK data through innovative trial design and careful consideration of PK sampling, as well as data from Ig treatment-naive patients with MMN, may further improve the understanding of dose–response relationships.

Our analysis is the first published that comprehensively characterizes the PK of IgG following IVIG 10% administration in patients with MMN using a fit-for-purpose model. This model offers an important tool for data analysis in a rare condition where limited data are available. Our analysis has provided insights into the kinetic interplay between endogenous and exogenous IgG, the impact of body size, and influential predictors for interindividual variability in the patient population. This model can serve as a framework for evaluation of IgG PK in subpopulations of interest in MMN or similar neuroimmunological indications, such as CIDP.

In conclusion, the findings of this study underscore the importance of stable dosing and consistent serum IgG levels in MMN to avoid negative responses owing to IVIG treatment interruptions, which often necessitate increased doses on resuming treatment. Dosing intervals more frequent than Q4W may alleviate periodic symptom deterioration by providing more stable and consistent IgG levels. In addition, dose splitting potentially offers flexibility for patients requiring large volumes of IVIG without negatively affecting serum IgG PK, while maintaining treatment efficacy. Our findings have important clinical relevance and offer guidance for physicians on optimizing and individualizing IVIG treatment in real-world clinical settings, as well as balancing convenience and dosing frequency in individual patients, especially with respect to symptom management.

## Data Availability

The data analyzed in this study is subject to the following licenses/restrictions: the data that support the findings of this study are available from the corresponding author upon reasonable request. Requests to access these datasets should be directed to Zhaoyang Li, zhaoyang.li@takeda.com.
