# Supplementary material for: Dose, exposure, and treatment regimen of intravenous immunoglobulin G in multifocal motor neuropathy
Source: Front Neurol. 2024 Nov 6;15:1478419. doi: 10.3389/fneur.2024.1478419 (PMC11580011; doi:10.3389/fneur.2024.1478419)
Supplement: Supplementary file 1 [file Data_Sheet_1.docx]

**SUPPLEMENTARY MATERIAL**

**Dose, exposure and treatment regimen of intravenous immunoglobulin G in multifocal motor neuropathy**

**Table of Contents**

[Supplementary table 1. Baseline patient characteristics and clinical covariates included in the PK data set. 2](#_Toc180679668)

[Supplementary results 5](#_Toc180679669)

[Supplementary references 10](#_Toc180679670)

#

# Supplementary table 1. Baseline patient characteristics and clinical covariates included in the PK data set.

| Patient characteristic | | Treatment sequence 1 (IVIG then placebo)^a^  (*n* = 22) | Treatment sequence 2 (placebo then IVIG)^a^  (*n* = 22) | Overall  (*N* = 44) |
| --- | --- | --- | --- | --- |
| Age, years | Mean (SD)  Median (min, max) | 51.8 (9.2)  52.0 (34.0, 72.0) | 51.6 (11.5)  50.0 (31.0, 70.0) | 51.7 (10.3)  52.0 (31.0, 72.0) |
| Weight, kg | Mean (SD)  Median (min, max) | 81.3 (14.3)  82.4 (56.3, 107.0) | 83.9 (11.1)  85.7 (65.1, 106.0) | 82.6 (12.7)  83.5 (56.3, 107.0) |
| BMI, kg/m^2^ | Mean (SD)  Median (min, max) | 27.7 (4.0)  26.1 (22.0, 37.3) | 28.0 (4.3)  27.7 (21.3, 39.1) | 27.9 (4.1)  27.3 (21.3, 39.1) |
| LBM, kg | Mean (SD)  Median (min, max) | 53.8 (8.3)  54.4 (40.3, 66.0) | 56.4 (5.3)  56.2 (47.0, 64.9) | 55.1 (7.0)  55.4 (40.3, 66.0) |
| IgG concentration, g/L | Mean (SD)  Median (min, max) | 20.7 (5.6)  19.4 (11.6, 31.8) | 21.1 (6.9)  21.1 (11.7, 37.0) | 20.9 (6.2)  20.2 (11.6, 37.0) |
| Total dose, g/kg | Mean (SD)  Median (min, max) | 1.0 (0.5)  0.9 (0.4, 2.0) | 1.1 (0.5)  1.0 (0.4, 2.0) | 1.0 (0.5)  1.0 (0.4, 2.0) |
| CrCL, mL/min | Mean (SD)  Median (min, max) | 121.6 (33.7)  119.7 (79.2, 195.5) | 114.2 (24.4)  114.7 (64.2, 165.9) | 117.9 (29.3)  117.5 (64.2, 195.5) |
| Race, *n* (%) | White  Black  Hispanic | 21 (95.5)  0  1 (4.5) | 20 (90.9)  2 (9.1)  0 | 41 (93.2)  2 (4.5)  1 (2.3) |
| Sex, *n* (%) | Male  Female | 13 (59.1)  9 (40.9) | 19 (86.4)  3 (13.6) | 32 (72.7)  12 (27.3) |
| Treatment interval, *n* (%) | Q2W  Q3W  Q4W | 4 (18.2)  2 (9.1)  16 (72.7) | 4 (18.2)  7 (31.8)  11 (50.0) | 8 (18.2)  9 (20.5)  27 (61.4) |
| Splitting scheme, *n* (%) | 1 day  2 days  3 days | 15 (68.2)  6 (27.3)  1 (4.5) | 13 (59.1)  6 (27.3)  3 (13.6) | 28 (63.6)  12 (27.3)  4 (9.1) |

^a^Treatment sequences refer to active treatment (IVIG 10% or placebo) during the crossover phases.

BMI, body mass index; CrCL, creatinine clearance; IgG, immunoglobulin G; IVIG 10%, intravenous immunoglobulin containing 10% IgG; LBM, lean body mass; PK, pharmacokinetic; Q2W, every 2 weeks; Q3W, every 3 weeks; Q4W, every 4 weeks; SD, standard deviation.

# Supplementary results

**Figure.** Goodness of fit plots for the final popPK model showing no significant deviation trends


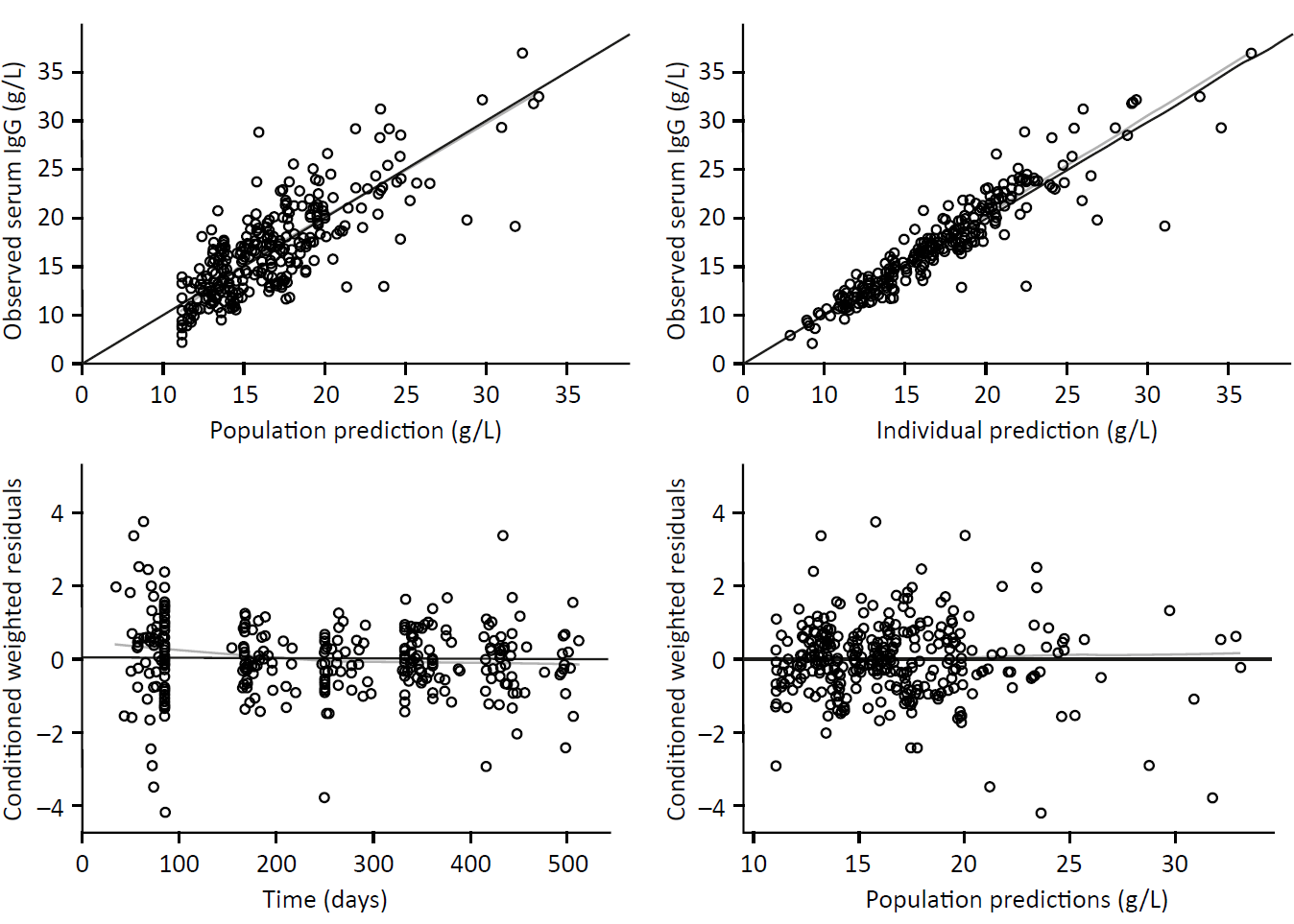


Dots represent individual data points, gray lines represent smoothed curve based on the data points, and black lines represent lines of identity or residuals equal zero.

IgG, immunoglobulin G; popPK, population pharmacokinetic.

**Figure.** VPCs for the final popPK model, stratified by treatment sequence


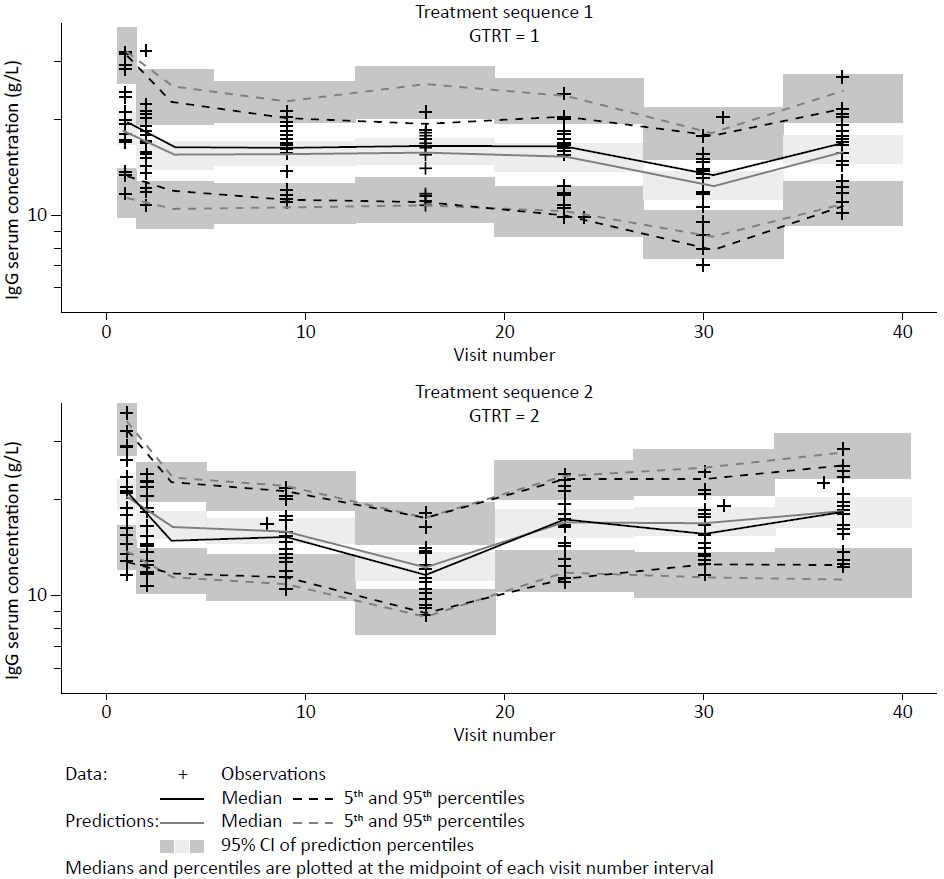


The VPC approach used NONMEM (supplemental references 1 and 2), and VPCs were based on 300 replicates of the analysis data set, that contained an overall population of 44 patients, or 22 patients per stratum. VPCs indicated a good fit of the central tendency and variability of the predicted IgG PK, with the observed data matching the prediction intervals for both the stabilization phases and the placebo phases.

CI, confidence interval; GTRT, coding of the treatment sequence (1 = IVIG then placebo; 2 = placebo then IVIG); IgG, immunoglobulin G; IVIG, intravenous immunoglobulin; PBO, placebo; popPK, population pharmacokinetic; VPC, visual predictive check.

**Individual exposure prediction**

To assess the clinical relevance of the model features and covariates, individual exposure parameters were predicted at steady-state and at the end of the placebo phase, stratified by treatment sequence and sex. Overall, PK parameters related to dosing or dose-normalized exposure were comparable between treatment sequences, and serum total IgG levels were also similar between men and women, as shown in the following table.

**Table.** Individual parameters related to dosing and IgG exposure, stratified by study arm and sex.

| Variable |  | Treatment sequence 1^a^  (*n* = 22) | Treatment sequence 2^a^  (*n* = 22) | Male  (*n* = 32) | Female  (*n* = 12) | Overall  (*n* = 44) |
| --- | --- | --- | --- | --- | --- | --- |
| Body weight-based dose, g/kg | Mean (SD)  Geo. mean (%CV)  Median (min, max) | 0.95 (0.48)  0.84 (53.62)  0.87 (0.40, 2.00) | 1.08 (0.51)  0.97 (51.20)  0.98 (0.40, 2.00) | 1.07 (0.53)  0.95 (54.30)  0.98 (0.40, 2.00) | 0.87 (0.38)  0.80 (46.46)  0.87 (0.40, 1.54) | 1.01 (0.49)  0.90 (52.34)  0.95 (0.40, 2.00) |
| Duration of placebo, days | Mean (SD)  Geo. mean (%CV)  Median (min, max) | 49.07 (31.59)  38.69 (87.08)  38.05 (7.00, 112.10) | 34.21 (27.23)  25.54 (92.24)  22.10 (7.00, 84.00) | 40.45 (32.57)  28.71 (105.26)  23.00 (7.00, 112.10) | 45.33 (23.61)  40.45 (51.92)  42.00 (21.00, 86.00) | 41.81 (30.14)  31.59 (92.62)  28.00 (7.00, 112.10) |
| CBASE, g/L | Mean (SD)  Geo. mean (%CV)  Median (min, max) | 11.52 (1.85)  11.37 (17.14)  12.00 (8.02, 14.04) | 11.26 (2.01)  11.10 (17.22)  11.26 (8.46, 16.80) | 11.66 (1.89)  11.52 (16.28)  11.79 (8.70, 16.80) | 10.67 (1.87)  10.52 (17.86)  10.74 (8.02, 13.93) | 11.39 (1.91)  11.23 (17.02)  11.63 (8.02, 16.80) |
| V1, L | Mean (SD)  Geo. mean (%CV)  Median (min, max) | 5.71 (2.05)  5.34 (39.63)  5.82 (2.67, 9.70) | 6.52 (1.43)  6.38 (21.93)  6.48 (4.24, 9.70) | 6.80 (1.43)  6.66 (20.60)  6.48 (4.81, 9.70) | 4.28 (1.34)  4.10 (31.54)  4.01 (2.67, 6.84) | 6.12 (1.79)  5.84 (32.79)  5.95 (2.67, 9.70) |
| CL, L/h | Mean (SD)  Geo. mean (%CV)  Median (min, max) | 0.01 (0.01)  0.01 (39.63)  0.02 (0.01, 0.02) | 0.02 (0.00)  0.02 (21.93)  0.02 (0.01, 0.02) | 0.02 (0.00)  0.02 (20.60)  0.02 (0.01, 0.02) | 0.01 (0.00)  0.01 (31.54)  0.01 (0.01, 0.02) | 0.02 (0.01)  0.02 (32.79)  0.02 (0.01, 0.02) |
| C_trough,ss_, g/L | Mean (SD)  Geo. mean (%CV)  Median (min, max) | 15.95 (3.59)  15.55 (24.14)  16.55 (9.87, 23.70) | 15.88 (3.27)  15.56 (20.86)  15.55 (10.50, 21.70) | 16.18 (3.67)  15.76 (24.09)  16.50 (9.87, 23.70) | 15.20 (2.48)  15.01 (17.01)  15.40 (10.70, 19.70) | 15.92 (3.39)  15.55 (22.28)  16.10 (9.87, 23.70) |
| C_trough,plc_, g/L | Mean (SD)  Geo. mean (%CV)  Median (min, max) | 12.40 (2.76)  12.08 (24.50)  13.10 (6.98, 17.50) | 12.37 (3.04)  12.04 (23.97)  11.75 (8.75, 18.10) | 12.73 (3.04)  12.36 (25.23)  12.60 (6.98, 18.10) | 11.54 (2.22)  11.34 (20.03)  11.30 (7.79, 15.30) | 12.39 (2.86)  12.06 (23.95)  12.35 (6.98, 18.10) |
| AUC_wk,ss_, g.h/L | Mean (SD)  Geo. mean (%CV)  Median (min, max) | 3529.4 (784.68)  3436.2 (24.88)  3759 (1850, 4645) | 3653.2 (903.32)  3548.7 (25.13)  3585 (2358, 5209) | 3590.7 (896.23)  3477.6 (26.70)  3585 (1850, 5209) | 3582.4 (694.18)  3518.9 (20.13)  3759 (2558, 4645) | 3588.3 (835.12)  3489.4 (24.74)  3680 (1850, 5209) |
| pAUC_wk.plc_, g.h/L | Mean (SD)  Geo. mean (%CV)  Median (min, max) | 2155.0 (411.25)  2115.7 (20.19)  2183 (1361, 2981) | 2152.4 (537.38)  2091.6 (24.77)  2139 (1513, 3215) | 2248.9 (482.21)  2198.4 (22.11)  2217 (1513, 3215) | 1916.0 (349.77)  1886.0 (18.91)  1942 (1361, 2472) | 2153.8 (469.52)  2104.2 (22.20)  2176 (1361, 3215) |
| C_avg,ss_, g/L | Mean (SD)  Geo. mean (%CV)  Median (min, max) | 21.01 (4.67)  20.45 (24.88)  22.37 (11.0, 27.7) | 21.75 (5.38)  21.12 (25.13)  21.34 (14.0, 31.0) | 21.37 (5.34)  20.70 (26.70)  21.34 (11.0, 31.0) | 21.32 (4.13)  20.95 (20.13)  22.37 (15.2, 27.7) | 21.36 (4.97)  20.77 (24.74)  21.91 (11.0, 31.0) |
| C_avg,plc_, g/L | Mean (SD)  Geo. mean (%CV)  Median (min, max) | 12.83 (2.45)  12.59 (20.19)  13.00 (8.10, 17.74) | 12.81 (3.20)  12.45 (24.77)  12.73 (9.01, 19.14) | 13.39 (2.87)  13.09 (22.11)  13.20 (9.01, 19.14) | 11.41 (2.08)  11.23 (18.91)  11.56 (8.10, 14.71) | 12.82 (2.80)  12.53 (22.20)  12.95 (8.10, 19.14) |

^a^Treatment sequences refer to active treatment (IVIG or placebo) during the crossover phases.

%CV, coefficient of variation expressed as a percent; AUC_wk_,_ss_, area under the concentration–time curve values for 1 week at steady-state during active treatment; C_avg,plc_, average serum concentration during the placebo period; C_avg,ss_, average serum concentration during the dosing interval at steady-state; CBASE, latent model-derived parameter of IgG concentration in the absence of treatment; CL, clearance; C_trough,plc_, serum concentration at trough during the placebo period; C_trough,ss_, serum concentration at trough at steady-state; Geo, geometric; IgG; immunoglobulin G; Max, maximum; Min, minimum; pAUC_wk,plc_, partial area under the concentration–time curve over the last week of the placebo period; SD, standard deviation; V1, volume of distribution.

# Supplementary references

1. NONMEM Nonlinear Mixed Effects Modelling Tool [Computer Program]. Icon Plc., Hanover, NH, USA. Available from: https://www.iconplc.com/solutions/technologies/nonmem.

2. Bergstrand M, Hooker AC, Wallin JE, Karlsson MO. Prediction-Corrected Visual Predictive Checks for Diagnosing Nonlinear Mixed-Effects Models. *AAPS J* (2011)
